# Seasonal and spatial dynamics of in-channel water bodies on a river sandbar and associated macroinvertebrate communities

**DOI:** 10.1371/journal.pone.0360065

**Published:** 2026-11-11

**Authors:** Moe Nakamura, Naoko Hara, Chiho Yamanaka, Hye-Ji Oh, Hideyuki Doi, Aki Tanaka, Yuko Ishida, Yasuhiro Takemon, Izumi Katano

**Affiliations:** 1 Graduate School of Humanities and Sciences, Nara Women’s University, Nara, Japan; 2 Organization for the Promotion of Gender Equality, Nara Women’s University, Nara, Japan; 3 Faculty, Division of Natural Science, Nara Women’s University, Nara, Japan; 4 Graduate School of Informatics, Kyoto University, Kyoto, Japan; 5 KYOUSEI Science Center, Nara Women’s University, Nara, Japan; 6 Faculty of Liberal Arts, Sciences and Global Education, Osaka Metropolitan University, Osaka, Japan; 7 Faculty of Science and Engineering, Setsunan University, Neyagawa, Japan; 8 Disaster Prevention Research Institute, Kyoto University, Uji, Japan; University of Zagreb Faculty of Teacher Education, CROATIA

## Abstract

In-channel water bodies (ICWBs) on river sandbars, including backwaters and riverine ponds shaped by hydrological disturbance, remain poorly understood with respect to their seasonal and fine-scale spatial dynamics. We investigated the environmental conditions and benthic macroinvertebrate assemblages in ICWBs formed around traditional wood-based river training structures (“Seigyu”) within a single sandbar in a regulated river in central Japan. Environmental conditions showed marked seasonal variation and fine-scale spatial gradients across habitat types over distances of tens of meters. Water temperature emerged as one of the strongest environmental gradients, exhibiting seasonal variation and longitudinal spatial structure within the sandbar. Macroinvertebrate communities in ICWBs were distinct from those in the adjacent main channel, and many taxa were restricted to ICWBs. Community composition varied among habitat types and across seasons, indicating that temporal processes interact with fine-scale spatial heterogeneity to structure assemblages. These results suggest that ICWBs on sandbars function as dynamic habitat patches whose ecological roles are shaped by the interaction between seasonal hydrological dynamics and fine-scale spatial heterogeneity.

## Introduction

Significant declines in freshwater biodiversity are a major global conservation concern, driven by multiple anthropogenic stressors including hydrological alteration, habitat fragmentation, and channel modification [1,2]. These declines are partly driven by the separation of main channels from floodplains through reduced flood pulses and lateral connectivity [3–5], although riverine ecosystems consist of interacting main-channel and adjacent habitats [6]. Such reductions in connectivity are caused not only by altered flow regimes associated with dam operations but also by reduced sediment transport resulting from channel incision and riverbed excavation [7,8]. Consequently, habitat heterogeneity within river channels has been simplified, leading to the degradation or loss of lentic and semi-lentic habitats.

Recent studies have therefore emphasized the importance of small-scale habitat units within river channels, such as backwaters and riverine ponds, because these habitats can create locally distinct environmental conditions within a single reach [9–11]. This perspective is consistent with the riverine ecosystem synthesis framework, which emphasizes the hierarchical organization and ecological significance of habitat patches within river channels [11]. In contrast to floodplain water bodies (FWBs), which typically develop on high-elevation alluvial terraces and experience relatively infrequent disturbance [12–14], in-channel water bodies (ICWBs) occur at lower elevations within the channel and are more directly influenced by channel-scale hydrological and geomorphological processes. ICWBs include side channels, backwaters partially connected to the main channel, and riverine ponds that are isolated from the main channel except during floods [9,15].

Here, we focus specifically on backwaters and riverine ponds formed on a single sandbar, whereas side channels are not considered because they represent hydrologically and geomorphologically distinct habitat types. Because ICWBs differ from both the main channel and FWBs in flow conditions, disturbance regimes, and substrate characteristics, they may support distinct and temporally variable aquatic communities that are not captured by reach-scale assessments. Benthic macroinvertebrates are suitable for evaluating such habitat heterogeneity because they respond sensitively to local environmental conditions and integrate spatial and temporal variability at fine scales [16]. However, while the ecological roles of side channels have been relatively well studied [e.g., 17, 18], macroinvertebrate communities in ICWBs remain poorly understood.

Beyond their geomorphological position, FWBs and ICWBs also differ in their disturbance regimes. FWBs generally experience relatively infrequent flood disturbance and often support well-developed riparian vegetation, whereas ICWBs are exposed to more frequent hydrological disturbance and channel-scale geomorphic processes [5]. Previous studies have shown that variation in hydrological connectivity and disturbance frequency influences both environmental conditions and the movement, colonization, and persistence of aquatic organisms [10,15,19,21,22]. However, most empirical studies have focused on FWBs or side channels, whereas ICWBs have received comparatively little attention [cf. 23]. Moreover, ICWBs within a single sandbar are unlikely to function as uniform habitat units: their ecological roles may vary with geomorphological position and seasonal hydrological conditions, yet this has rarely been examined explicitly. Accordingly, how environmental and spatial gradients within ICWBs interact with seasonal processes to shape benthic macroinvertebrate communities remains poorly understood, particularly at the scale of individual sandbars.

In ICWBs, backwater and riverine ponds are formed in locally depressed areas on sandbars that are inundated during floods, often through bed scour occurring near flow obstructions such as boulders, large woody debris, or channel structures [24]. These geomorphic processes can generate a mosaic of lentic and semi-lentic habitats within a single sandbar, resulting in substantial small-scale environmental heterogeneity. In central Japan, traditional wood-based river training structures known as “Seigyu” have been used to influence local flow patterns and sediment dynamics [25] (Fig 1c, d). Beyond bank protection, Seigyu guide sediment deposition and maintain channel complexity by promoting the formation of complex ICWBs, which has motivated their recent reintroduction in restoration-oriented river management [26]. Seigyu consist of timber-frame structures installed on sandbars to induce localized scour and sediment deposition during flood events, thereby promoting the formation of backwaters and riverine ponds following inundation [26]. Previous studies have shown that ICWBs formed around Seigyu are hydrologically dynamic and exhibit spatial variation in environmental conditions within the sandbar [24]. Although Seigyu-associated ICWBs provide a useful system for examining habitat differentiation within a sandbar, it remains unclear how seasonal hydrological dynamics and fine-scale spatial heterogeneity jointly structure benthic macroinvertebrate communities [e.g., 27].

**Fig 1:**
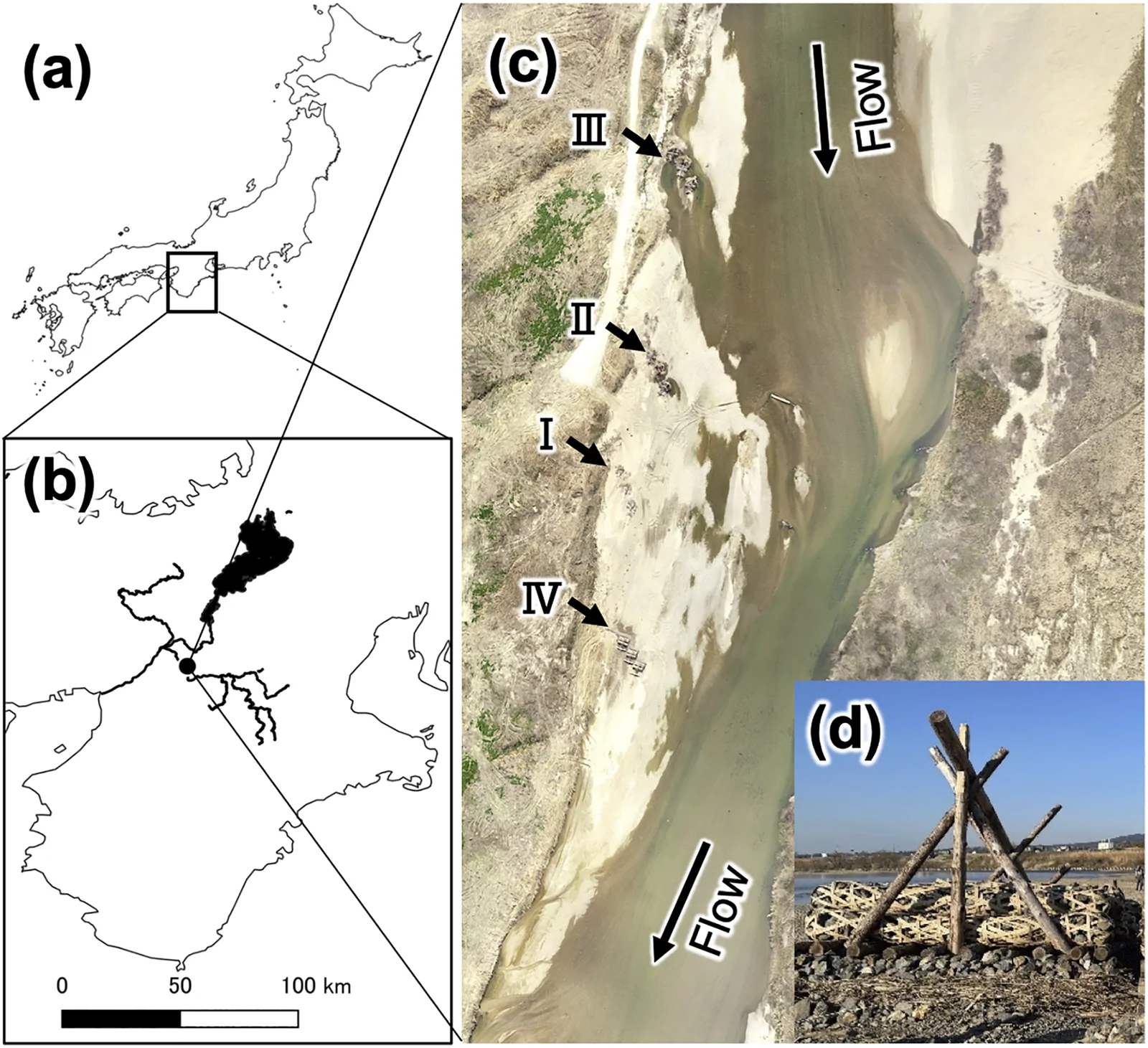
Sampling sites. Map of Japan (a), the Yodo River system (b), the sampling sites on the right bank of the Kizu River (c), and a representative Seigyu unit (d). In (c), a drone photograph shows the sandbar located 15.2 km upstream from the confluence. Arrows indicate Seigyu groups (each consisting of three Seigyu units; I-IV), constructed on December 16, 2017 (I), December 16, 2018 (II), November 24, 2019 (III), and December 6, 2020 (IV).

Given the formation of diverse ICWBs on sandbars by traditional Seigyu structures [24], a key unresolved question is how environmental and spatial heterogeneity within these ICWBs is reflected in biological community structure. Although ICWBs have the potential to enhance habitat heterogeneity within river channels [11,20], the environmental and spatial factors associated with community variation remain insufficiently understood. In particular, it is unclear how local environmental conditions (e.g., water quality and substrate characteristics) and spatial factors (e.g., position within a sandbar) are associated with benthic macroinvertebrate communities in ICWBs. It also remains unclear how these relationships vary seasonally. Seasonal disturbance and dispersal processes may further influence these relationships [28,29]. In addition, because in-channel lentic habitats are often assumed to function as refugia [30,31], it is important to test whether riverine ponds on sandbars support persistent assemblages or instead undergo repeated seasonal reorganization. Clarifying this point is necessary not only for understanding the ecological roles of ICWBs themselves, but also for evaluating how they may complement, rather than replace, the functions of FWBs in regulated rivers. To address these gaps, the objectives of this study were to: (1) describe spatial and seasonal variation in environmental conditions among different types of ICWBs (i.e., backwaters and riverine ponds (RPs)) formed around Seigyu within a single sandbar, (2) characterize benthic macroinvertebrate community structure in these ICWBs relative to a nearby main-channel habitat, and (3) examine how environmental and spatial gradients within the sandbar are associated with variation in macroinvertebrate community composition.

## Methods

We conducted seasonal field sampling of environmental conditions and benthic macroinvertebrate communities in backwaters and riverine ponds (hereafter, RP), which together represent in-channel water bodies (ICWBs), on a single sandbar in the lowland reach of the Kizu River, central Japan. The study focused on ICWBs formed around traditional wooden river-training structures (“Seigyu”) and was designed to characterize spatial and seasonal variation in environmental conditions, differences in macroinvertebrate community structure between ICWBs and a nearby main-channel reference, and associations between environmental gradients and community composition within the sandbar. Field sampling was conducted on five occasions from June 2020 to June 2021, following the installation of Seigyu structures that had been implemented annually since 2017.

### Study site

The Kizu River is a major tributary of the Yodo River system, which flows into Osaka Bay, Japan. The river originates in Mie Prefecture, flows through Kyoto Prefecture, and joins the Katsura and Uji Rivers near the border between Kyoto and Osaka Prefectures to form the Yodo River (Fig 1a, b). The Kizu River is approximately 99 km long and has a basin area of 1,663 km^2^. In the upper reaches, several multipurpose dams constructed between the late 1960s and the late 1990s have resulted in regulated flow regimes [4], while the weathered granite geology of the basin provides abundant sediment that promotes sandbar development in the lowland reaches, including the study site.

The study site was located in the lowland reach of the Kizu River, approximately 15.2 km upstream from the confluence with the Yodo River (34°48’27.1” N, 135°48’10.9” E, Fig 1b, c). At this site, traditional wooden river-training structures known as “Seigyu” have been installed annually from 2017 on a sandbar as part of a river management initiative aimed at modifying local flow and sediment dynamics during flood events (Fig 1c, d). Seigyu consist of timber-frame units placed within woven bamboo baskets and weighted with boulders to prevent displacement during high flows [25]. Although Seigyu historically played an important role in river management in Japan, their use declined during the mid-20th century as river engineering shifted toward standardized concrete-based flood-control structures designed for efficient bank stabilization and channel regulation [25]. Beyond bank protection, Seigyu were also traditionally used to guide sediment deposition and maintain channel complexity, which has motivated their recent reintroduction in restoration-oriented river management.

In the study reach (approximately 200–400 m with seasonal changes), Seigyu structures were installed as sets consisting of three units, with one set deployed annually during the winters of 2017, 2018, 2019, and 2020, resulting in a total of four sets (12 individual units; Fig 1c). Following flood disturbances, two types of ICWBs developed around the Seigyu structures: backwaters that maintained partial surface connections to the main channel, and RPs that remained isolated from the main channel except during high-flow events. Because Seigyu structures of different installation ages co-occurred within the study sandbar, the present study focused on spatial and seasonal habitat differentiation rather than explicitly evaluating installation-age effects. By spring of 2020, when the study began, multiple ICWBs of both types had already formed on the sandbar.

### Field sampling

We conducted field sampling on June 3, August 11, November 21, 2020, and on March 3 and June 1, 2021 (Fig 2). Precipitation and river water-level data were obtained from a Japanese Meteorological Agency station in Kyotanabe City (4.7 km from the study site) and from the Inooka gauge station, located approximately 1 km upstream. During the study period, several major flood disturbances occurred during the rainy season, whereas minor disturbances were observed intermittently outside winter (Fig 2). When water levels exceeded –2.0 m, some low-elevation RPs connected with the main channel, and when levels exceeded –1.5 m, all RPs on the sandbar became connected to the river.

**Fig 2:**
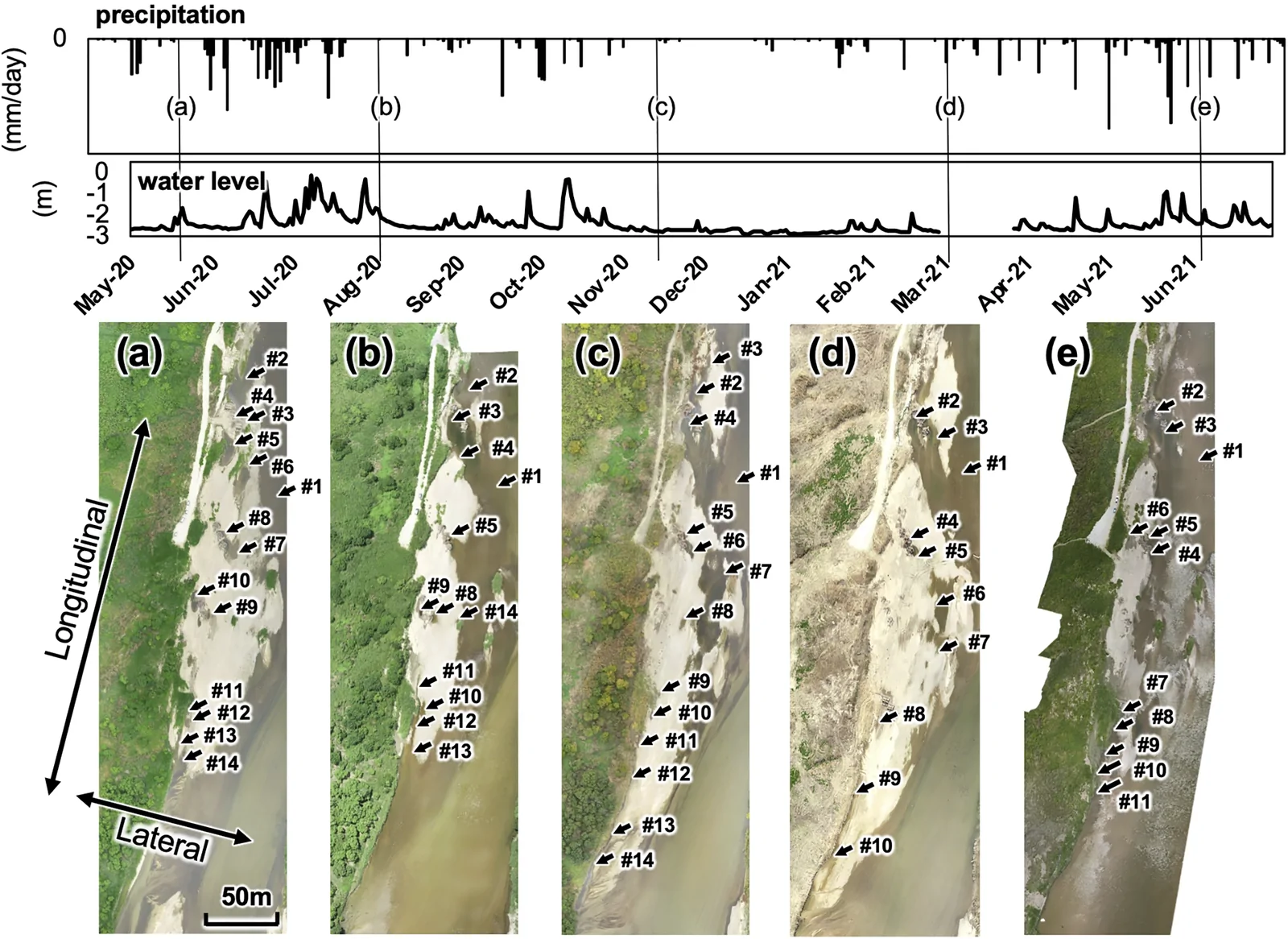
Precipitation, river water level, and drone photographs of the study sandbar. Daily precipitation data were obtained from the Japan Meteorological Agency. River water level data were obtained from the Hydrologic Quality Database of Ministry of Land, Infrastructure, Transport and Tourism; data were unavailable from 27 February to 3 April 2021. Vertical lines (a–e) indicate field sampling dates, corresponding to the drone photographs shown in panels (a-e), which were used to identify habitat configuration. Arrows and labels in the photographs indicate sampling points, including the main-channel reference site (#1). Double-headed arrows indicate the longitudinal (upstream–downstream) and lateral (channel–margin) directions within the sandbar.

We surveyed all ICWBs present on the sandbar in each season (8–14 sites per survey) and one adjacent main-channel site as a reference. Sampling units were defined as site-by-season observations, with habitat type (backwater or RP) determined at the time of each survey. A main-channel site was selected to provide a consistent reference for seasonal comparisons. Two backwaters were consistently present (upstream and downstream), and when sufficiently large, multiple sampling sites were established within a backwater to capture internal heterogeneity (Fig 2 and Table 1). Backwaters were classified as upstream backwaters (UB) or downstream backwaters (DB) based on their position relative to the main-channel flow direction, as these positions differ in hydraulic exposure and connectivity during flow fluctuations.

**Table 1:** Number of sampling sites.

	Date	Up-backwater	Down-backwater	Riverine ponds	Main channel
Number of sampling sites	Jun. 2020	1	2	10	1
	Aug. 2020	3	4	4	1
	Nov. 2020	3	3	7	1
	Mar. 2021	2	1	6	1
	Jun. 2021	2	2	6	1
					
Detailed ID	Jun. 2020	#2	#13, 14	#3, 4, 5, 6, 7, 8, 9, 10, 11, 12	#1
	Aug. 2020	#2, 3, 4	#10, 11, 12, 13	#5, 8, 9, 14	#1
	Nov. 2020	#2, 3, 4	#12, 13, 14	#5, 6, 7, 8, 9, 10, 11	#1
	Mar. 2021	#2, 3	#10	#4, 5, 6, 7, 8, 9	#1
	Jun. 2021	#2, 3	#10, 11	#4, 5, 6, 7, 8, 9	#1

Numbers with the symbol ‘#’ correspond to the ICWB numbers in Fig 2. When UBs and DBs exceeded approximately 100 m in length, multiple sampling sites were established to capture environmental heterogeneity within each backwater.

ICWB locations were identified using drone imagery (Mavic 2, DJI, Shenzhen, China). The latitude and longitude at each sampling site were recorded using a GPS unit (WGPS2, Meruem, Shizuoka, Japan), and water depth at the deepest point was measured using a graduated scale. Water temperature (°C), electrical conductivity (mS m^-1^), and pH were measured in surface water using an EC meter (CT-27112B, TOA DKK, Tokyo, Japan) and a pH meter (Twin PH-11B LAQUA, Horiba Ltd., Kyoto, Japan). All measurements for a given sampling were completed within 20 min to minimize temporal variation among sampling sites.

To estimate phytoplankton biomass, chlorophyll *a* (Chl-*a*) concentrations were determined from 4 L of surface-water samples collected at the center of each ICWB. To quantify fine particulate organic and inorganic matter (FPOM, including zooplankton, and FPIM), 8–80 L of surface water were filtered through two overlaid plankton nets (mesh sizes: 1 mm and 100 μm). Filtered volumes were adjusted among sites to obtain sufficient material while avoiding clogging. Material retained on the 100 µm mesh (i.e., particles <1 mm and >100 µm) was stored in 250 mL polypropylene bottles and transported to the laboratory under refrigerated conditions.

Benthic macroinvertebrates were semi-quantitatively sampled in each ICWB using a D-frame net (mesh size: 500 µm, opening: 30 × 30 cm). Two replicate D-frame samples were consistently collected for 30 s each at every sampling site, regardless of habitat size, using a standardized time-based sampling protocol, and pooled into a single composite sample for each site-by-season observation. When large backwaters contained multiple sampling sites, this procedure was repeated independently at each sampling site. This standardized time-based approach enabled consistent comparisons of macroinvertebrate assemblages among sites and seasons [e.g., 32, 33], while acknowledging that absolute density estimates may vary with substrate conditions and sampling efficiency. Sampling was conducted along a transect encompassing the range of microhabitats from the center to the edge of each ICWB. Immediately after collection, all samples were preserved in the field with formalin to a final concentration of 10% for subsequent laboratory analysis.

The authors confirm that this study complied with the current laws of Japan. Field surveys were conducted with permission from the Yodogawa River Office, Kinki Regional Development Bureau, Ministry of Land, Infrastructure, Transport and Tourism, Japan. Because no fish sampling or collection was conducted, no special collection permit was required under current Japanese regulations.

### Laboratory analyses

To quantify the spatial position and occupied area of each ICWB on the sandbar, the longitudinal distance (m) from the upstream edge of the sandbar, the lateral distance (m) from the river edge, and the occupied area (m^2^) were measured using ImageJ (NIH) based on drone imagery.

To measure chlorophyll a (Chl-*a*) concentration as an index of phytoplankton biomass, water samples were gently mixed and filtered within 24 h of collection through a glass-fiber filter (GF/F; GE Healthcare, Chicago, USA). Filters were placed in vials containing 8 mL of 99.5% ethanol and stored in the dark at 4 ºC for 24 h. Pigments were extracted and quantified using a spectrophotometer (U-1800; Shimadzu Co., Kyoto, Japan) following [34].

To determine fine particulate organic matter (FPOM) and fine particulate inorganic matter (FPIM) concentrations, samples collected using plankton nets were filtered through GF/F filters. Filters were dried at 60 °C for 48 h and weighed using an electronic balance (AW220; Shimadzu Co., Kyoto, Japan), then combusted at 550 °C for 2 h in a muffle furnace (FO610; Yamato Scientific Co., Ltd., Tokyo, Japan), reweighed, and used to calculate ash-free dry mass (AFDM) and ash mass. FPOM and FPIM concentrations (mg m^-3^) were calculated by dividing AFDM and ash mass by the filtered water volume.

To assess water quality, dissolved inorganic nitrogen (nitrate, NO_3_^-^) and soluble reactive phosphorus (SRP) concentrations were measured. Water samples were filtered through pre-combusted GF/F filters. Nitrate concentration was determined following [35–37], and SRP concentration was analyzed using the ascorbic acid molybdenum blue method [38].

For macroinvertebrate analysis, samples preserved in formalin were rinsed with tap water and sieved through a 1 mm mesh. Individuals ≥1 mm were retained for analysis to ensure consistent taxonomic identification and comparability among habitat samples, because mesh size and inclusion of small early instars can influence assemblage characterization and identification consistency [e.g., 39]. Accordingly, individuals between 500 µm and 1 mm were not included in the community analyses. Macroinvertebrates were sorted, counted, and identified to the lowest possible taxonomic level (usually to genus) using the identification keys of [40–42].

### Statistical analyses

All statistical analyses were performed using R language (ver. 4.4.2; [43]). An alpha level of 0.05 was used where significance testing was applied. Sampling units were defined as site-by-season observations, and site identity was included where appropriate. To address the study objectives, complementary analyses were used to examine (1) spatial and seasonal variation in environmental variables, (2) differences in macroinvertebrate community structure between ICWBs and the main-channel reference, and (3) associations between environmental gradients and community composition. Unless otherwise noted, analyses were used to describe patterns rather than to infer causal relationships.

(1) **Environmental variation within ICWBs**

To describe spatial and seasonal variation in environmental variables within ICWBs, generalized linear mixed-effects models (GLMMs) were applied to site-by-season observations from individual sampling sites within the sandbar. At each survey, habitats were classified as upstream backwaters (UB), downstream backwaters (DB), or riverine ponds (RP) according to observed habitat conditions. Because habitat configuration varied seasonally, some sampling sites differed in habitat category among surveys. Environmental variables included water depth, occupied area, water temperature, pH, electrical conductivity, chlorophyll a concentration, fine particulate organic matter (FPOM), and nutrient concentrations (soluble reactive phosphorus and nitrate nitrogen). Habitat type (UB, DB, or RP), season, and their interaction were included as fixed effects, and site identity was included as a random effect. Environmental variables were analyzed using Gaussian generalized linear mixed-effects models with an identity link, and model assumptions were evaluated using residual diagnostics. The significance of fixed effects was evaluated using likelihood-ratio χ² tests. These models were used to summarize patterns in environmental variation rather than for strict inferential testing.

To examine spatial patterns within the sandbar, simple linear regressions and generalized additive models (GAMs) were conducted between environmental variables and spatial position along longitudinal (upstream–downstream) and lateral (channel–margin) axes. Based on Shapiro-Wilk normality tests, EC, chl.a, and FPOM were log_10_-transformed. Analyses were performed separately for each season. Linear regressions were used as the primary analyses, whereas generalized additive models (GAMs) were used as complementary analyses to evaluate whether major spatial patterns deviated substantially from linear relationships.

(2) **Community comparisons with the main-channel reference**

Differences in macroinvertebrate community metrics between ICWBs and the main channel were evaluated using effect sizes for total abundance, taxon richness, and Shannon diversity. For each ICWB site and season, log response ratios (LRR) were calculated as LRR = log(X_ICWB / X_main-channel), where X represents each benthic metric. LRRs were interpreted as relative contrasts to the single main-channel reference and used to visualize differences across seasons.

To examine differences in the composition of habitat-specific and shared taxa between ICWBs and the main channel, taxon occurrence patterns were quantified for each site and season. Taxa were classified as (i) common taxa (occurring in both the main channel and ICWBs), (ii) main-channel-only taxa, or (iii) ICWB-only taxa. For each category, proportions were calculated as the number of taxa in that category divided by the total number of taxa observed at each site and season. These occurrence data were analyzed using binomial GLMMs based on the numbers of taxa belonging and not belonging to each category, with habitat category (main channel, UB, DB, or RP), season, and their interaction as fixed effects and site identity as a random effect. The significance of fixed effects was evaluated using likelihood-ratio χ^2^ tests. Model assumptions were evaluated using residual diagnostics, and post hoc pairwise comparisons of habitat categories were performed using estimated marginal means with Tukey adjustment for multiple comparisons, where appropriate.

(3) **Multivariate community analyses within ICWBs**

Multivariate analyses of macroinvertebrate community composition were conducted using the “vegan” package (ver. 2.7-2; [44]). Differences in community composition among ICWB habitat types and seasons were assessed using permutational multivariate analysis of variance (PERMANOVA) based on Bray–Curtis dissimilarity of Hellinger-transformed abundance data using the adonis2 function in the “vegan” package. To account for repeated observations of the same sampling sites across seasons, permutations were restricted within site identity. PERMANOVA was applied only to ICWB samples, while main-channel samples were used as a reference for visualization. Prior to PERMANOVA, homogeneity of multivariate dispersion was evaluated using PERMDISP. Non-metric multidimensional scaling (NMDS) was used to visualize assemblage patterns using the metaMDS function in the “vegan” package.

Similarity percentage (SIMPER) analysis based on Bray–Curtis dissimilarity was used as a descriptive tool to identify taxa contributing to within-habitat similarity and between-habitat dissimilarity, thereby aiding interpretation of observed multivariate patterns.

Redundancy analysis (RDA) was conducted using the rda function in the “vegan” package to examine relationships between environmental gradients and macroinvertebrate community composition. Species abundance data were Hellinger-transformed prior to RDA, and environmental variables were screened for multicollinearity using variance inflation factors (VIF), with highly correlated variables excluded. The significance of the overall RDA model and individual axes was evaluated using permutation tests, and results were interpreted as exploratory patterns rather than as evidence of causal relationships.

Rarefaction curves were generated to assess sampling completeness within each season and were interpreted descriptively. Variation partitioning analysis was conducted using the varpart function in the “vegan” package to explore the relative contributions of environmental and spatial factors to community variation. Results are presented in S2 Fig in the Supplementary Material and were treated as hypothesis-generating rather than confirmatory.

## Results

The numbers and spatial distribution of ICWBs varied among seasons during the study period (Table 1 and Fig 2a–e). A variety of ICWB categories, including UBs, DBs, and RPs, were observed on the sandbar throughout the study period (Fig 3). The extent and internal heterogeneity of backwater habitats changed seasonally, resulting in variation in the number of sampling points within UB and DB among seasons. In contrast, each RP was represented by a single sampling site.

**Fig 3:**
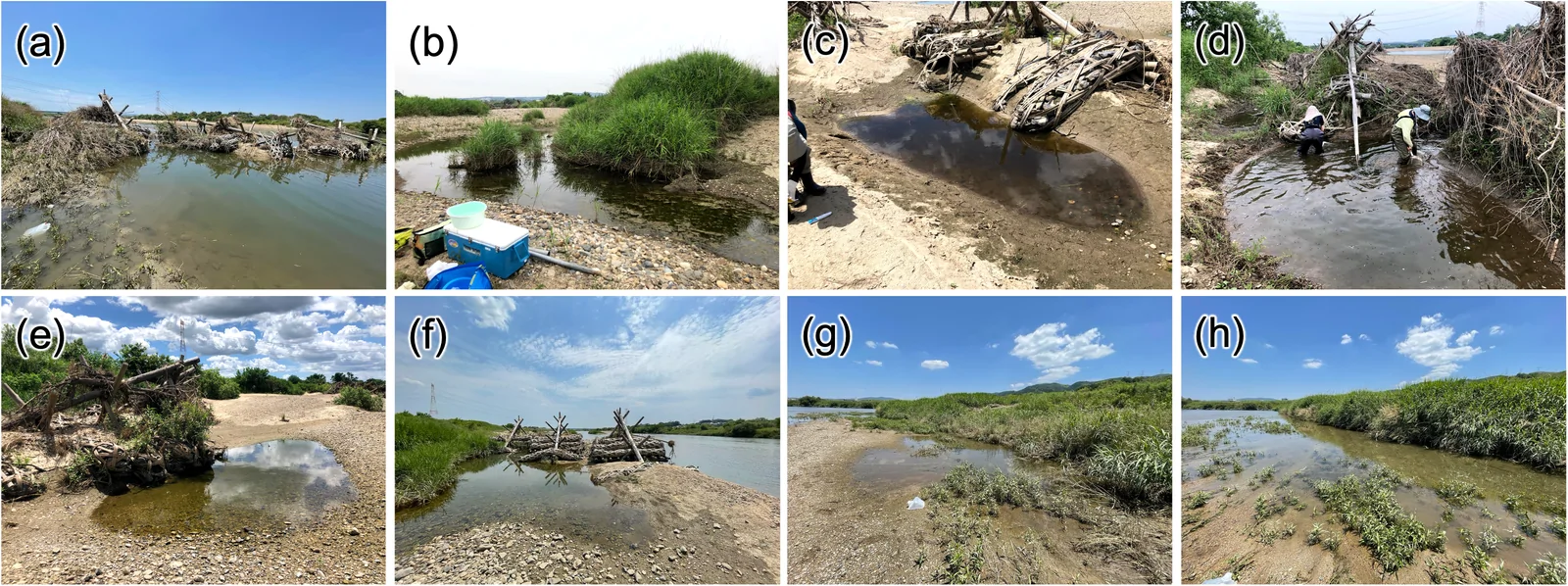
Representative photographs of the in-channel water bodies (ICWBs) during the study period. (a) Upstream backwater (#3) in August 2020, (b) riverine pond (#5) in June 2020, (c) riverine pond (#9) in June 2020, (d) riverine pond (#10) in June 2020, (e) riverine pond (#8) in August 2020, (f) riverine pond (#7) in June 2021, (g) downstream backwater (#10) in June 2021, and (h) downstream backwater (#11) in June 2021.

Environmental variables measured in the ICWBs are summarized in Table 2. Five variables, including area, water temperature, pH, PO_4_-P, and NO_3_-N, showed pronounced seasonal variation (GLMM, χ^2^ = 10.94, 289.82, 11.45, 41.00, and 166.52, respectively). Water temperature and NO_3_-N varied among habitat categories (χ^2^ = 14.42 and 13.61, respectively). Seasonal variation also differed among habitat categories for area, water temperature, pH, PO_4_-P, and NO_3_-N (χ^2^ = 3.92, 9.91, 10.54, 13.65, and 9.61, respectively). Seasonal patterns were generally consistent across ICWB categories. Area exhibited substantial seasonal variability, while water temperature and pH were generally higher in summer and lower in winter. In contrast, PO_4_-P and NO_3_-N concentrations tended to be lower in summer and higher in winter. Differences among habitat categories were also evident, although these patterns varied seasonally. Water temperature tended to be relatively high in RPs during summer and in DBs during winter (except June 2021), whereas lower values were generally observed in DBs during June 2020 and June 2021 and UBs during winter and August 2020. NO_3_-N concentrations were generally higher in both UBs and DBs than in RPs, except in March 2021.

**Table 2:** Environmental variables for each survey date and site category.

	20-Jun	20-Aug	20-Nov	21-Mar	21-Jun	GLMM	*χ2*	*P*	
Depth (cm)									
Up-backwater	20.0 (1)	71.3 ± 56.75 (3)	62.8 ± 14.15 (3)	58.8 ± 17.32 (2)	61.5 ± 12.02 (2)	habitat type	0.32	0.575	
Down-backwater	8.0 ± 2.82 (2)	10.8 ± 2.63 (4)	20.0 ± 0 (3)	19.0 (1)	27.8 ± 3.89 (2)	season	2.65	0.449	
Riverine ponds	32.3 ± 18.59 (10)	19.5 ± 7.14 (4)	28.8 ± 19.07 (7)	29.1 ± 21.72(6)	39.4 ± 19.91 (6)	habitat type*season	2.97	0.396	
Main channel	25.00 (1)	17.0 (1)	30.5 (1)	30.5 (1)	43.0 (1)				
Area (m^2^)									
Up-backwater	360.0 (1)	797.0 ± 0 (3)	1453.9 ± 0 (3)	1272.4 ± 0 (2)	340.6 ± 0 (2)	habitat type	2.05	0.152	
Down-backwater	167.6 ± 0 (2)	283.4 ± 0 (4)	102.4 ± 0 (3)	487.7 (1)	56.8 ± 0 (2)	season	10.94	0.012	*
Riverine ponds	36.4 ± 42.65 (10)	8.2 ± 7.26 (4)	150.7 ± 367.31 (7)	243.9 ± 430.40 (6)	87.8 ± 64.73 (6)	habitat type*season	3.92	0.27	
Main channel	n.a.	n.a.	n.a.	n.a.	n.a.				
Water temperature (°C)									
Up-backwater	25.7 (1)	28.6 ± 0.57 (3)	16.2 ± 0.70 (3)	8.5 ± 0.07 (2)	21.3 ± 0.21 (2)	habitat type	14.42	0.0001	***
Down-backwater	19.1 ± 5.44 (2)	29.0 ± 1.73 (4)	16.9 ± 0.40 (3)	11.4 (1)	18.2 ± 0.28 (2)	season	289.82	<0.00001	***
Riverine ponds	25.8 ± 1.97(10)	31.6 ± 2.93 (4)	16.7 ± 1.17 (7)	9.8 ± 0.87 (6)	20.6 ± 5.44 (6)	habitat type*season	9.91	0.0193	*
Main channel	25.0 (1)	29.2 (1)	15.8 (1)	8.9 (1)	20.9 (1)				
pH									
Up-backwater	9.1 (1)	8.0 ± 0.35 (3)	8.3 ± 0.47 (3)	8.0 ± 0.07 (2)	7.5 ± 0.14 (2)	habitat type	0.92	0.337	
Down-backwater	9.0 ± 0.49 (2)	7.8 ± 0.70 (4)	7.6 ± 0.72 (3)	7.4 (1)	7.2 ± 0.14 (2)	season	11.45	0.0095	**
Riverine ponds	8.7 ± 0.66 (10)	8.3 ± 0.65 (4)	9.0 ± 0.78 (7)	7.7 ± 0.08 (6)	7.3 ± 0.14 (6)	habitat type*season	10.54	0.0145	*
Main channel	9.2 (1)	7.3 (1)	8.6 (1)	8.3 (1)	7.3 (1)				
EC (mS m^-1^)									
Up-backwater	14.0 (1)	12.6 ± 0.11 (3)	13.3 ± 0.10 (3)	13.6 ± 0.01 (2)	12.3 ± 0.09 (2)	habitat type	1.66	0.198	
Down-backwater	12.8 ± 0.71 (2)	11.3 ± 0.26 (4)	12.0 ± 1.06 (3)	13.3 (1)	13.1 ± 0.04 (2)	season	6.67	0.083	
Riverine ponds	14.1 ± 1.19 (10)	13.9 ± 2.68 (4)	17.7 ± 9.24 (7)	13.5 (6)	14.6 ± 4.70 (6)	habitat type*season	7.32	0.0625	
Main channel	14.0 (1)	12.4 (1)	14.1 (1)	13.5 (1)	12.2 (1)				
chl. *a*. (mg m^3^)									
Up-backwater	36.1 (1)	15.8 ± 1.63 (3)	4.6 ± 1.79 (3)	5.4 ± 2.12 (2)	2.3 ± 0.36 (2)	habitat type	0.5	0.478	
Down-backwater	78.7 ± 97.69 (2)	55.0 ± 46.30 (4)	16.8 ± 12.38 (3)	1.6 (1)	0.7 ± 0.61 (2)	season	0.93	0.817	
Riverine ponds	108.4 ± 204.80 (10)	42.9 ± 29.05 (4)	30.9 ± 31.14 (7)	3.4 ± 1.84 (6)	10.2 ± 22.40 (6)	habitat type*season	0.31	0.959	
Main channel	12.8 (1)	19.6 (1)	4.5 (1)	4.4 (1)	1.9 (1)				
FPOM (mg L^-1^)									
Up-backwater	1010.0 (1)	395.5 ± 363.65 (3)	1641.8 ± 894.99(3)	262.9 ± 43.42 (2)	1.0 ± 0.05 (2)	habitat type	0.84	0.359	
Down-backwater	490.4 ± 11.68 (2)	1891.0 ± 2188.29 (4)	918.0 ± 523.85(3)	529.6 (1)	0.9 ± 0.13 (2)	season	0.48	0.923	
Riverine ponds	2229.6 ± 2549.34 (10)	1000.5 ± 834.53 (4)	3202.6 ± 3429.59 (7)	1258.5 ± 1475.41 (6)	0.8 ± 0.28 (6)	habitat type*season	1.69	0.639	
Main channel	324.0 (1)	40.2 (1)	121.6 (1)	127.4 (1)	137.4 (1)				
PO_4_-P (µmol L^-1^)									
Up-backwater	0.2 (1)	0.4 ± 0.03 (3)	1.0 ± 0.02 (3)	1.0 ± 0.05 (2)	0.98 ± 0.05 (2)	habitat type	0.42	0.518	
Down-backwater	0.3 ± 0.30 (2)	0.3 ± 0.16 (4)	0.8 ± 0.07 (3)	0.9 (1)	0.86 ± 0.13 (2)	season	41	<0.00001	***
Riverine ponds	0.2 ± 0.14 (10)	0.7 ± 0.57 (4)	0.6 ± 0.28 (7)	1.0 ± 0.11 (6)	0.81 ± 0.28 (6)	habitat type*season	13.65	0.0034	**
Main channel	0.4 (1)	0.4 (1)	0.9 (1)	1.0 (1)	1.04 (1)				
NO_3_-N (µmol L^-1^)									
Up-backwater	24.1 (1)	33.9 ± 2.38 (3)	53.58 ± 2.15 (3)	73.23 ± 0.34 (2)	54.94 ± 0.35 (2)	habitat type	13.61	0.0002	***
Down-backwater	29.0 ± 13.28 (2)	17.6 ± 4.85 (4)	52.78 ± 5.96 (3)	71.41 (1)	47.43 ± 2.50 (2)	season	166.52	<0.00001	***
Riverine ponds	13.0 ± 6.79 (10)	17.2 ± 4.67 (4)	39.65 ± 8.43 (7)	73.87 ± 1.10 (6)	42.97 ± 16.14 (6)	habitat type*season	9.61	0.0222	*
Main channel	33.7 (1)	37.5 (1)	56.93 (1)	72.74 (1)	54.11 (1)				

Means and standard deviations are shown, and the number of sites is given in parentheses. n.a., not applicable. Results of generalized linear mixed-effects models (GLMMs; site identity as a random effect) are shown as likelihood-ratio χ^2^ statistics and *P* values for habitat type (UB, DB, RP), season, and their interaction. *: *P* < 0.05.

Seasonal spatial patterns of environmental variables along the longitudinal and lateral gradients of the sandbar are shown in Fig 4a and b, respectively. Generalized additive models (GAMs) were also conducted as complementary non-linear analyses (S1 Fig). Because the GAM results were broadly consistent with the linear models, the linear regression results (Fig 4) are presented as the primary analyses. However, because spatial autocorrelation was not explicitly modeled, these analyses should be interpreted as descriptive evaluations of directional spatial gradients within the sandbar. Several environmental variables showed season-dependent relationships with spatial position along both gradients. Along the longitudinal gradient, relationships with spatial position were observed for five variables (Fig 4a). Electrical conductivity (EC) showed negative relationships from upstream to downstream in June 2020 and March 2021, whereas water temperature showed a positive longitudinal relationship in March 2021. pH showed a negative relationship in March 2021, chlorophyll *a* showed positive and negative relationships in June 2020 and March 2021, respectively, and NO_3_-N showed a negative relationship in August 2020. Along the lateral gradient, EC showed a positive relationship in June 2021, chlorophyll a showed a positive relationship in August 2020, FPOM showed a positive relationship in June 2020, and NO_3_-N showed a negative relationship in August 2020.

**Fig 4:**
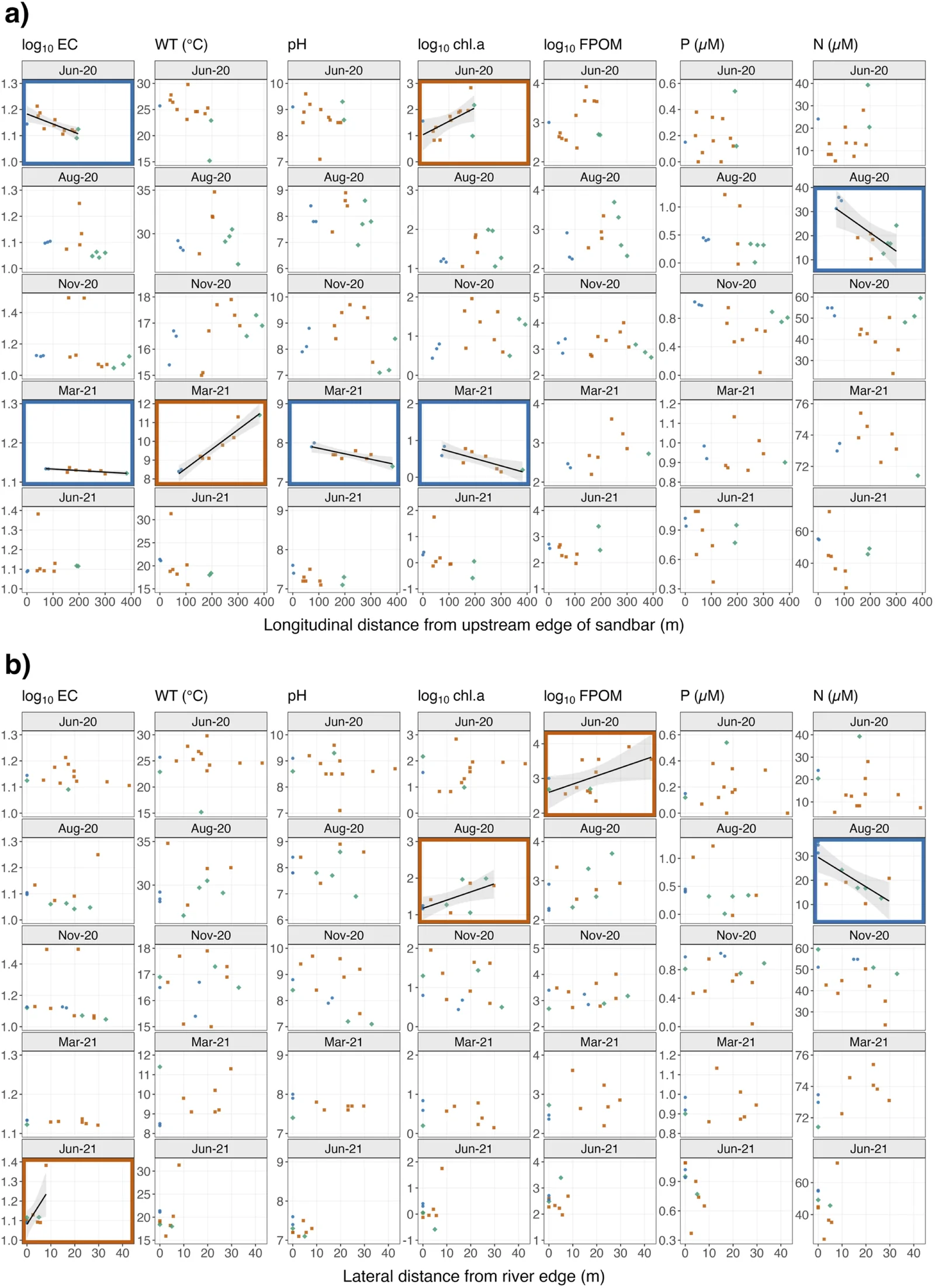
Longitudinal and lateral gradient of environmental variables within ICWBs on the sandbar. Results of simple linear regressions against longitudinal distance from the upstream edge of the sandbar (a) and lateral distance from the water’s edge (b). EC, chl. *a* and FPOM were log_10_-transformed based on the results of Shapiro-Wilk normality tests. For variables showing significant relationships (*P* < 0.05), positive slopes are indicated by red boxes and negative slopes by blue boxes. Symbols and colors indicate ICWB categories: upstream backwater (circle and blue), riverine pond (square and orange), and downstream backwater (diamond and green).

LRRs are shown to illustrate relative differences between ICWBs and the main-channel reference across seasons (Fig 5). These LRRs quantify the relative deviation of ICWB communities from the main-channel reference for each site-by-season observation. LRRs showed marked variation among seasons and habitat categories. For abundance, LRRs were generally positive in DBs, except in March 2021. Taxon richness showed relatively little variation among habitat categories and seasons. For Shannon diversity, LRRs tended to be higher in UBs than in DBs and RPs, with relatively higher values observed during autumn and winter than during summer.

**Fig 5:**
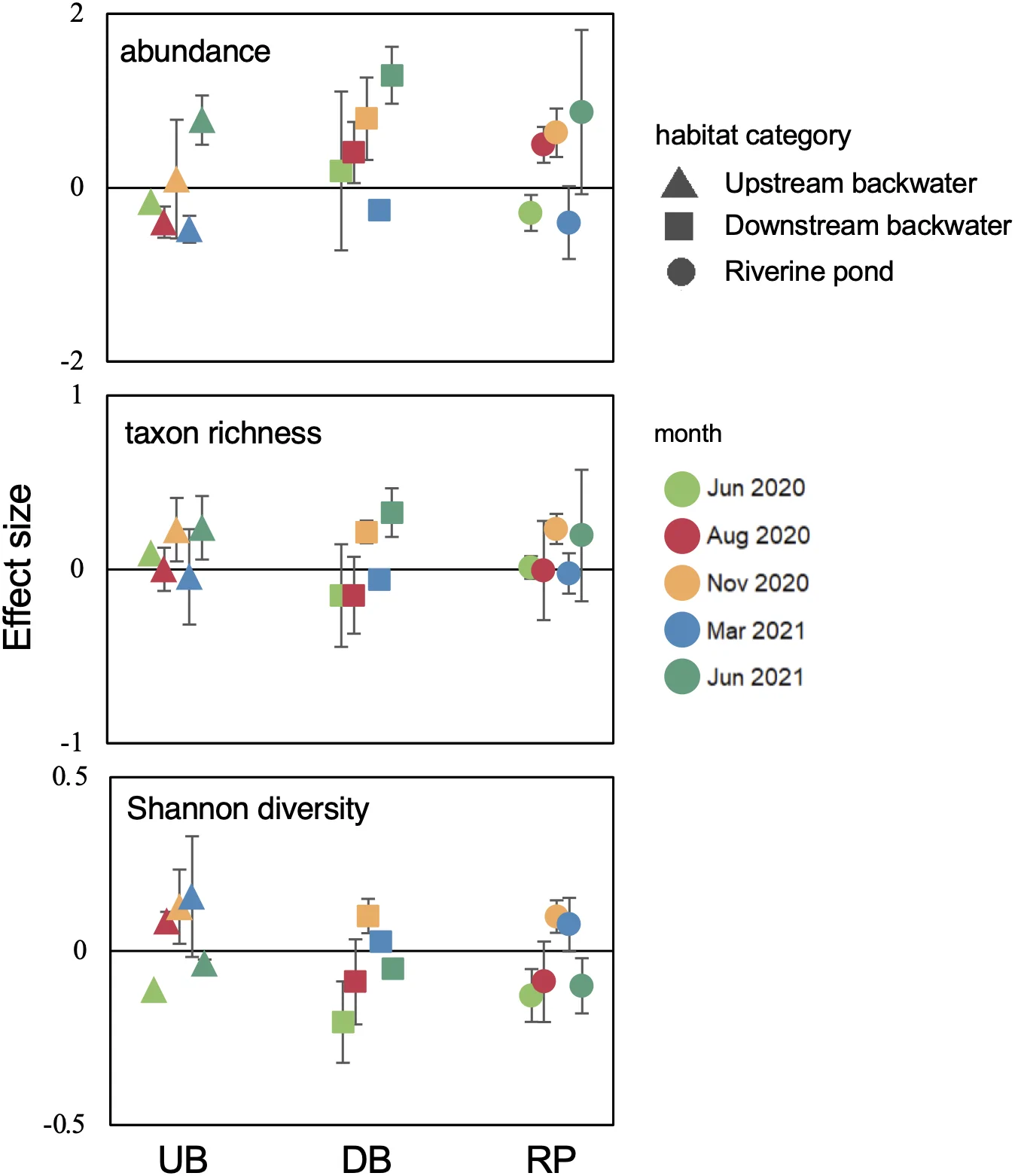
Effect sizes of macroinvertebrate metrics in ICWBs relative to the main channel reference site. Effect sizes were calculated as log-response ratios for three macroinvertebrate metrics (abundance, taxon richness, and Shannon diversity) in upstream backwaters (UB), downstream backwaters (DB), and riverine ponds (RP). Points show means and error bars indicate 95% confidence intervals. Colors indicate survey dates, and symbol shapes indicate ICWB categories.

The proportions of taxa shared between or restricted to ICWBs and the main channel are shown by season and site in Fig 6. Generalized linear mixed models (GLMMs), with site included as a random effect, indicated that neither season nor habitat category significantly affected the proportions of taxa in the three occurrence categories (GLMM, χ^2^ = 3.258 and 1.863, respectively). Most taxa observed in the main channel occurred in both the main channel and ICWBs, whereas taxa occurring exclusively in the main channel were few. In contrast, approximately half of the taxa observed in ICWBs were restricted to ICWBs. The proportion of habitat-restricted taxa was significantly higher in ICWBs than in the main channel (post hoc pairwise comparison with Tukey adjustment, *P* < 0.05), whereas no significant seasonal differences were detected (χ^2^ = 4.658).

**Fig 6:**
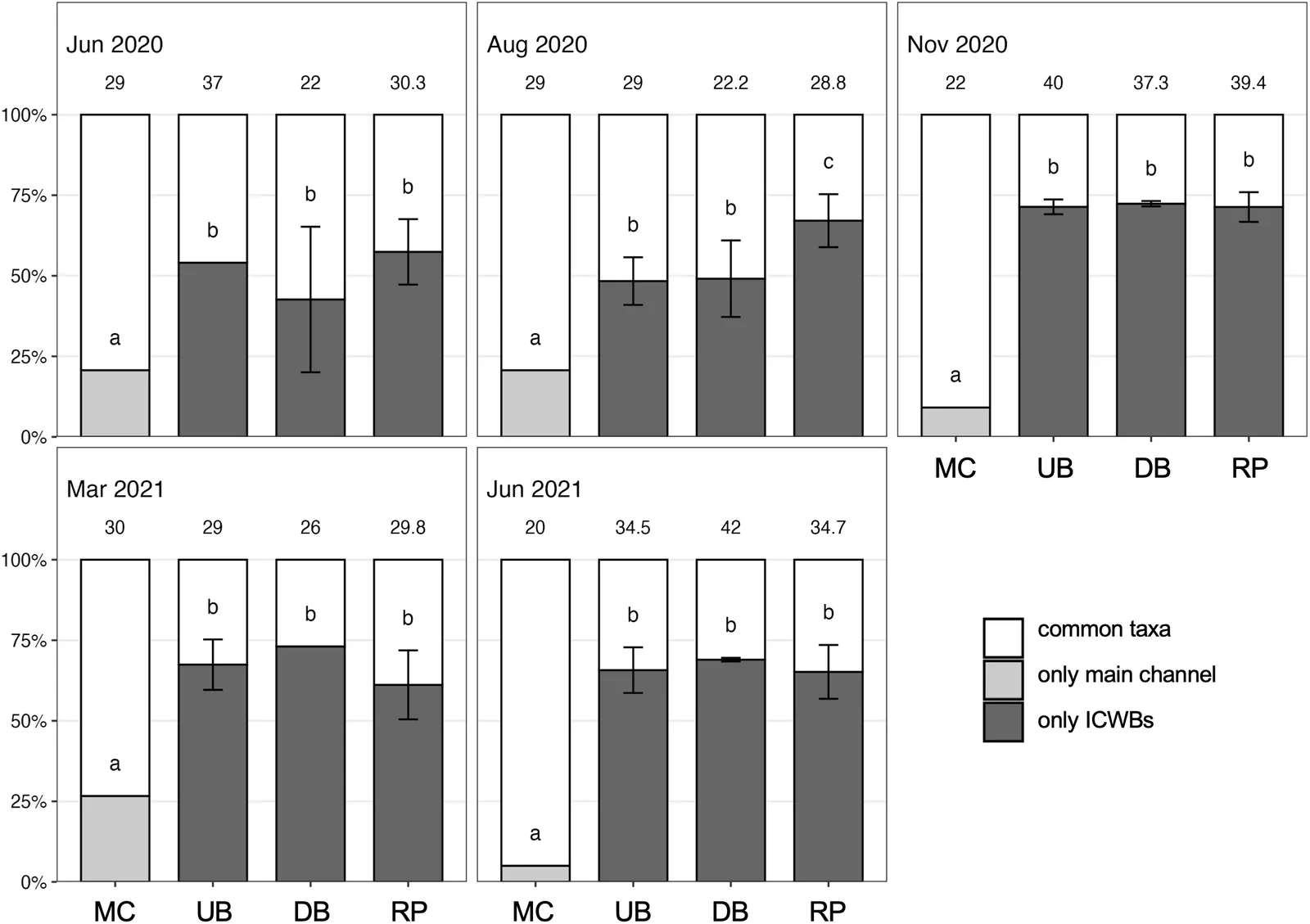
Proportional composition of macroinvertebrate taxa among site categories for each survey date. Bars show the percentage of observed taxa in each site category. Numbers above bars indicate mean taxon richness. Light gray, dark gray, and open bars represent taxa occurring only in the main channel, only in ICWBs, and in both habitat types, respectively. Different letters indicate significant differences among site categories based on post hoc pairwise comparisons with Tukey adjustment following generalized linear mixed-effects models (GLMMs, *P* < 0.05). Horizontal lines indicate means, and error bars indicate standard deviations.

Differences in macroinvertebrate community composition among habitat categories and seasons are summarized in Table 3. PERMDISP detected significant differences in multivariate dispersion among habitat categories but not among seasons (*F* = 11.56 and 0.26, respectively). PERMANOVA revealed significant differences in community composition among habitat categories and seasons (*F* = 1.11 and 2.39, respectively; *P* = 0.001 for both), whereas the habitat category × season interaction was not significant (*F* = 1.22, *P* = 0.075; Table 3). Because dispersion differed among habitat categories, PERMANOVA results should be interpreted with caution, as they may partly reflect differences in within-group variability. NMDS ordination (stress = 0.23) provided a visual overview of seasonal and habitat-related community patterns (Fig 7). Across all seasons, macroinvertebrate communities in the main channel tended to be separated from those in ICWBs. Within ICWBs, UBs and DBs generally fell within the distributional range of RPs in most seasons, except in August 2020, when downstream backwaters were positioned closer to the main-channel assemblages. Ordination positions varied seasonally, with substantial overlap among June and August 2020 and June 2021.

**Table 3:** Results of PERMANOVA and PERMDISP testing for differences in macroinvertebrate community composition among ICWB habitat types and seasons.

	PERMANOVA		PERMDISP	
	*F*	*P*	*F*	*P*
habitat type	1.11	0.001**	11.56	<0.00001***
season	2.39	0.001**	0.26	0.901
habitat type*season	1.22	0.075		

Analyses were based on Bray–Curtis dissimilarity of Hellinger-transformed abundance data and were applied only to ICWB samples (UB, DB, RP); the main-channel site was used only as a reference in ordination (Fig 7). For PERMANOVA, permutations were restricted within site identity to account for repeated observations of the same sampling sites across seasons. *P < 0.05; **P < 0.01; ***P < 0.001.

**Fig 7:**
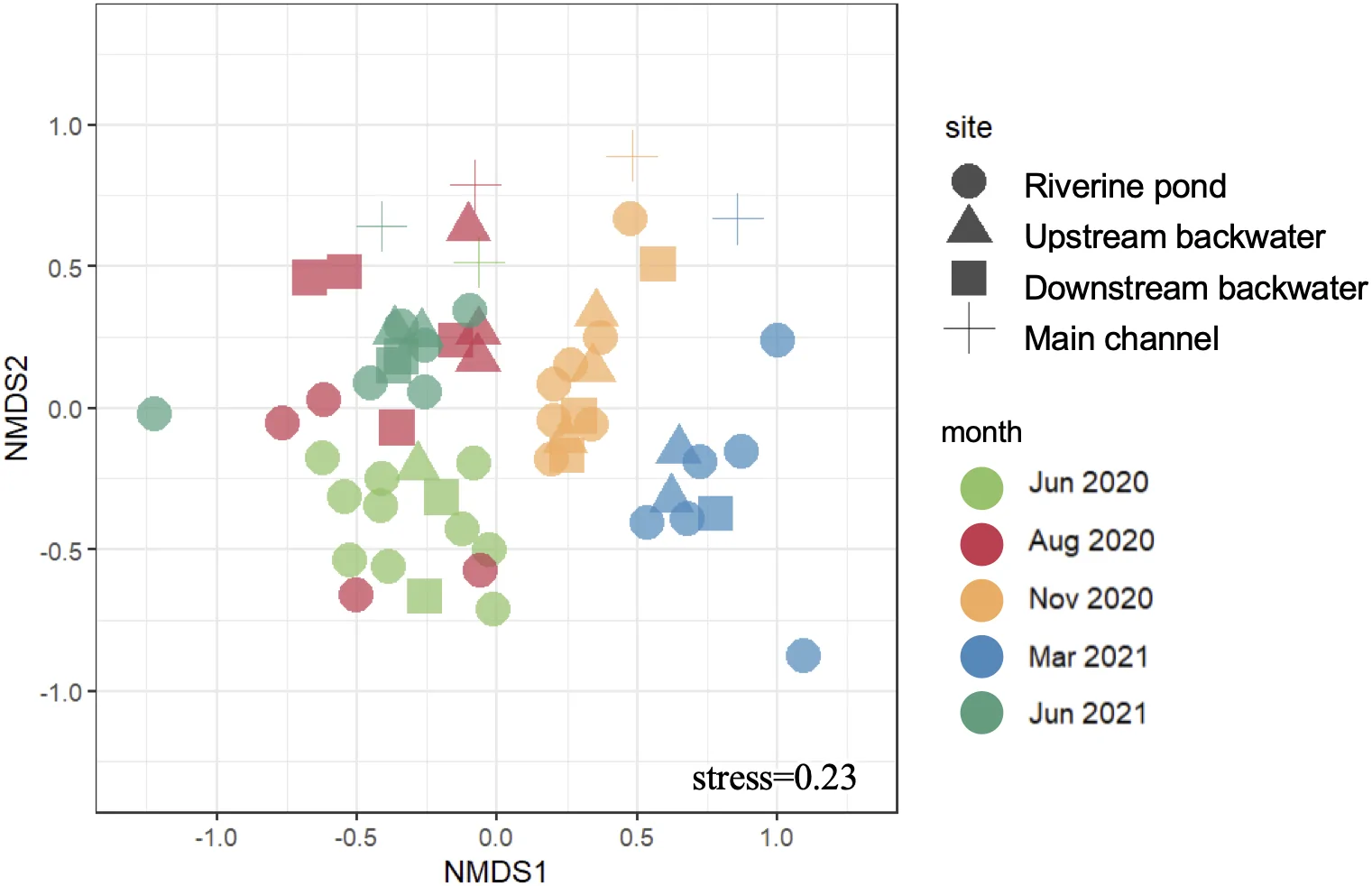
Non-metric multidimensional scaling (NMDS) ordination of macroinvertebrate communities. Colors indicate survey dates, and symbol shapes indicate site categories. Stress = 0.23; the ordination is presented for overview, with statistical inference based on PERMANOVA.

SIMPER analysis identified taxa contributing most to within-habitat similarity for each habitat category (Table 4). These taxa contributed most to within-group similarity and should not be interpreted as strict indicator taxa. In the pooled analysis across all seasons, no taxa met the criterion for contributing strongly to within-habitat similarity in RPs.

**Table 4:** Results of SIMPER analysis for each habitat category across all seasonal samples combined.

	Up-backwater	Down-backwater	Riverine ponds	Main channel
The number of extracted taxa	7	16	0	17
				
Rank				
1st	Gammaridae	Chironominae	ー	*Drunella ishiyamana*
2nd	*Labiobaetis* sp.	*Leptocerus* sp.	ー	*Rhithrogena japonica*
3rd	BIVALVIA	Veliidae	ー	*Acentrella gnom*
4th	Hydrophilidae	TURBELLARIA	*ー*	Athericidae
5th	*Ephemera orientalis*	Tipulidae	ー	Ceratopogonidae

SIMPER was conducted using Bray–Curtis dissimilarity to identify taxa contributing most to within-category similarity and between-category dissimilarity. The number of extracted taxa and the top five contributing taxa are shown.

RDA results for each season are shown in Fig 8 and Table 5. From June 2020 to June 2021, the cumulative proportion of variance explained by the first two axes ranged from 0.51 to 0.71, whereas the third axis explained only 0.10–0.17, indicating that the first two axes captured major gradients in community composition. Among environmental variables, water temperature ranked among the three longest vectors in four of five seasons (Table 5). Permutation tests indicated that the constrained ordination models were significant in all seasons (*P* < 0.05), with adjusted R^2^ values ranging from 0.21 to 0.39.

**Table 5:** Eigenvalues and environmental vector lengths of seasonal RDA.

		20-Jun			20-Aug			20-Nov			21-Mar			21-Jun		
		RDA1	RDA2	Vector length	RDA1	RDA2	Vector length	RDA1	RDA2	Vector length	RDA1	RDA2	Vector length	RDA1	RDA2	Vector length
	Eigenvalue	0.14	0.1	—	0.12	0.09	—	0.08	0.06	—	0.16	0.06	—	0.14	0.06	—
	Cumulative Proportion	0.42	0.71	—	0.36	0.62	—	0.3	0.51	—	0.4	0.56	—	0.5	0.71	—
	Biplot scores															
	Area	-0.28	-0.63	0.69	-0.65	0.1	**0.66**	-0.49	0.36	**0.61**	0.27	0.56	0.62	-0.37	0.36	0.52
	Lateral distance	-0.3	0.65	**0.72**	0.22	-0.28	0.35	0.43	-0.78	**0.9**	0.04	-0.22	0.23	0.68	-0.39	0.78
	Longitudinal distance	-0.26	-0.28	0.38	0.04	0.31	0.31	0.08	-0.23	0.24	0.17	-0.7	**0.72**	-0.18	-0.52	0.55
	Depth	-0.48	0.55	**0.73**	-0.2	-0.1	0.22	-0.2	0.57	0.6	-0.44	0.34	0.56	-0.48	0.69	**0.84**
	Water temperature	0.26	0.69	**0.74**	0.75	0.09	**0.76**	-0.62	-0.46	**0.77**	0.15	-0.6	0.62	0.9	-0.07	**0.9**
	pH	-0.09	-0.17	0.19	0.26	-0.05	0.26	-0.36	-0.09	0.37	0.22	0.71	**0.75**	0.13	0.17	0.21
	Chl. a	0.31	-0.22	0.38	0.18	0.21	0.28	-0.25	0.03	0.25	-0.29	0.5	0.57	0.96	-0.15	**0.97**
	FPOM	-0.11	0.34	0.35	0.31	0.32	**0.44**	-0.27	-0.52	0.59	0.71	-0.21	**0.74**	-0.16	-0.47	0.5

Species abundances were Hellinger-transformed prior to RDA, and environmental variables were screened for multicollinearity using variance inflation factors (VIF) before model fitting. Environmental factors with the maximum and minimum scores on RDA1 and RDA2 for each survey date are shown in red and blue, respectively. The top three environmental factors based on vector length are shown in bold.

**Fig 8:**
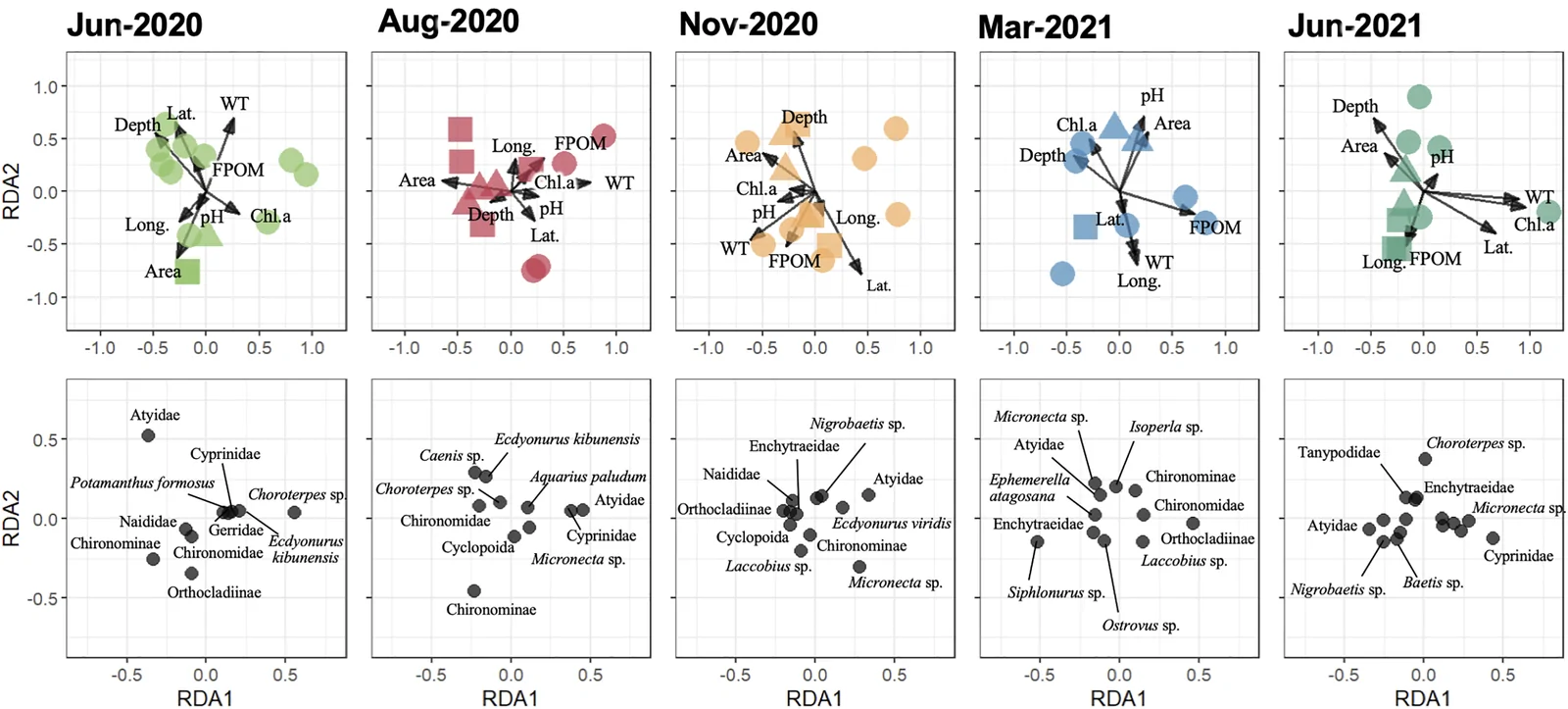
Redundancy analysis (RDA) biplots showing the relationships between environmental variables and sampling sites (upper panels), and macroinvertebrate taxa (lower panels) for each survey date. Environmental variables include lateral distance from water’s edge (Lat.), longitudinal distance from the upstream end of the sandbar (Long.), and water temperature (WT). Symbols indicate site categories: triangles (upstream backwater, UB), squares (downstream backwater, DB), and circles (riverine ponds). Colors indicate sampling events.

Rarefaction curves for macroinvertebrate communities in RPs are shown in Fig 9. In all seasons, rarefaction curves did not reach an asymptote, indicating that taxon accumulation was not saturated with the current sampling effort. Results of the exploratory variation partitioning analysis are presented in S2Fig. The unique fractions explained by environmental and spatial factors were 0.178 and 0.113, respectively, with a small shared fraction of 0.010.

**Fig 9:**
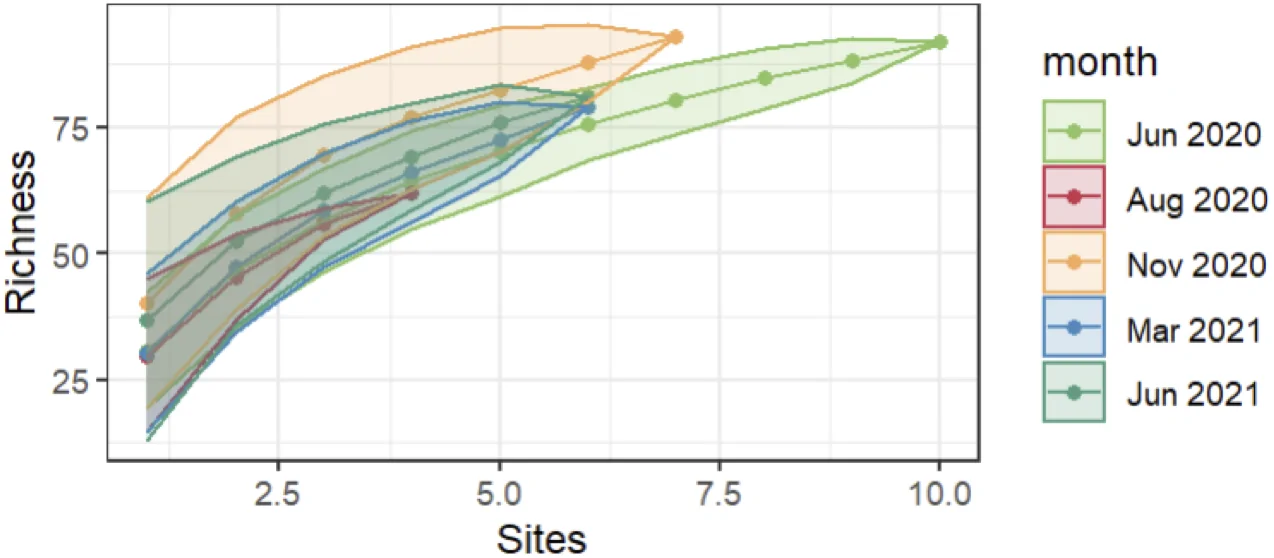
Sample-based rarefaction curves for macroinvertebrate taxa in riverine ponds. Curves are shown separately for each survey date.

## Discussion

### Environmental characteristics of in-channel water bodies

In-channel water bodies (ICWBs) formed on river sandbars were characterized by marked seasonal variation and fine-scale spatial heterogeneity in environmental conditions, indicating that they function as dynamic habitat patches rather than as uniform lentic units within the channel. In river–floodplain systems, temporal variation in water level and connectivity driven by flood pulses governs habitat conditions and material exchange [1,3]. Although ICWBs are commonly classified as lentic or semi-lentic habitats in river habitat typologies [e.g., 9, 15], their physical and chemical environments respond sensitively to seasonal changes in flow regime, sediment dynamics, and hydrological connectivity with the main channel [19,20]. Our results extend this concept by showing that pronounced environmental variability can emerge even within a single sandbar.

Within sandbars, ICWBs were further structured by fine-scale geomorphological gradients, reflected in differences in water temperature, nutrient concentrations, electrical conductivity, and habitat area among habitat types and seasons. UBs generally exhibited deeper and more thermally stable conditions, whereas DBs tended to be shallower and more depositional. RPs were characterized by relatively isolated and seasonally variable conditions, often associated with elevated summer water temperatures and variable nutrient concentrations depending on hydrological connectivity. Together, these findings indicate that environmental heterogeneity within ICWBs is structured by both seasonal hydrological dynamics and fine-scale geomorphological position, forming a heterogeneous habitat mosaic consistent with the riverine ecosystem synthesis framework [11]. Such habitat heterogeneity provides the environmental template underlying the differences in macroinvertebrate assemblages among UBs, DBs, and RPs described in the following sections.

### Seasonal variation and longitudinal temperature gradients within ICWBs

Seasonal variation strongly influenced the environmental conditions within ICWBs, with water temperature emerging as one of the most consistently structured variables across habitat types and seasons. Water temperature is widely recognized as a fundamental environmental factor governing physical processes and biological activity in river ecosystems [45,46], and in the present study it was generally higher in summer and lower in winter across all ICWB types. In addition to this seasonal pattern, water temperature exhibited distinct longitudinal gradients within the sandbar, indicating spatially structured thermal conditions even over distances of tens of meters.

Such fine-scale thermal variation has been linked to hyporheic exchange and subsurface flow paths within gravel and sandbar environments [47,48]. Notably, the direction of longitudinal temperature gradients differed among seasons, with downstream ICWBs tending to be cooler than upstream ones during summer but warmer during winter. Similar seasonal reversals in thermal gradients have been reported in systems where subsurface water exchange and residence time vary spatially and temporally [24,49].

These patterns suggest that thermal conditions within ICWBs are influenced not only by atmospheric forcing but also by local hydrological and geomorphological processes operating within the sandbar. Seasonal changes in hydrological connectivity and subsurface flow paths may alter the relative influence of surface and subsurface water inputs, contributing to the observed reversals in longitudinal temperature gradients [19,49–51]. Consequently, differences in subsurface water exchange and habitat-specific water residence time may provide an important environmental context for interpreting macroinvertebrate community structure within ICWBs.

### Macroinvertebrate community responses to environmental gradients

Macroinvertebrate community composition in ICWBs varied in response to the interaction between seasonal dynamics and fine-scale environmental gradients within the sandbar, rather than being structured by habitat category alone. Among the measured environmental variables, water temperature consistently represented one of the strongest gradients across seasons, whereas other physicochemical variables contributed in a season-dependent manner [sensu 11].

Seasonal variation accounted for a larger proportion of variation in community structure than habitat category in the PERMANOVA (R² = 0.151 vs. 0.035, respectively; Table 3), although the significant difference in multivariate dispersion among habitat categories warrants caution when interpreting the habitat effect. This pattern suggests that temporal processes, including hydrological disturbance and life-history dynamics, may contribute substantially to the seasonal reorganization of ICWB assemblages, while spatial heterogeneity within the sandbar may also contribute to differences in community composition among habitat categories.

Despite this strong seasonal influence, clear spatial differentiation was observed among UB, DB, and RP, reflecting habitat-specific environmental contexts within the sandbar. Although these ICWB types were spatially proximate, they differed in physical and chemical characteristics, including water temperature and nutrient concentrations, resulting in distinct community compositions.

Macroinvertebrate diversity was consistently higher in UBs than in DBs across seasons, suggesting that UBs provide relatively stable and heterogeneous conditions supporting a wider range of taxa. In contrast, DBs were characterized by assemblages associated with shallower, depositional, and more disturbance-prone environments. Such differentiation among adjacent ICWBs supports the view that fine-scale variation in hydrological connectivity and disturbance regimes within sandbars can generate distinct ecological niches over spatial scales of tens of meters [10,17].

RPs, in contrast, showed no taxa that were consistently shared across all seasons, indicating pronounced seasonal turnover in community composition. Taxa associated with RPs varied substantially among seasons, suggesting repeated community reassembly under changing environmental conditions rather than persistence of a stable core assemblage. Although ICWB assemblages were clearly distinct from those in the main channel and supported many habitat-restricted taxa, lotic taxa from the main channel occurred only intermittently. Together, these findings suggest that RPs contribute to biodiversity primarily through seasonal turnover and habitat heterogeneity rather than through the long-term persistence of refuge populations.

Given the close spatial proximity of ICWBs within the sandbar, dispersal limitation alone is unlikely to explain the observed patterns. Instead, community assembly within ICWBs appears to reflect the interaction of stochastic colonization, season-specific environmental filtering, and disturbance-driven resetting of local assemblages, allowing priority effects to influence subsequent community structure [28, 29].

### Integrated interpretation: spatial–temporal dynamics of ICWBs within the sandbar

The present study suggests that ICWBs on sandbars function as spatially heterogeneous and seasonally dynamic habitat components within river channels rather than as uniform or static habitat units. Their ecological roles were structured by the interaction between geomorphological position and seasonal hydrological variation, resulting in habitat differentiation over relatively small spatial scales [3,19,20].

Differences in macroinvertebrate community structure between UBs and DBs can be interpreted in the context of contrasting geomorphological settings. UBs generally represented deeper and hydraulically more stable habitats, whereas DBs developed under shallower and more depositional conditions [17,52].

These relatively stable upstream habitats supported taxa typically associated with stable substrates and longer developmental periods, such as *Ephemera japonica* and *Drunella ishiyamana* [41,53]. The consistently higher macroinvertebrate diversity observed in UBs further suggests that relatively stable habitats promote the coexistence of a broader range of taxa. In contrast, DBs supported assemblages dominated by opportunistic and deposit-associated taxa, including Chironominae and Naididae, which are commonly associated with fine-sediment and organic-rich environments [41,54]. Together, these patterns support the interpretation that fine-scale geomorphological heterogeneity contributes to functional differentiation among ICWBs, even over spatial scales of tens of meters, consistent with previous observations that geomorphological variation among riverine backwaters promotes differentiation in benthic macroinvertebrate communities [10]. Compared with DBs, UBs generally exhibited more stable environmental conditions and tended to support higher macroinvertebrate diversity. These observations suggest that UBs may contribute to maintaining local biodiversity through time, whereas DBs may contribute by promoting seasonal habitat heterogeneity and community turnover. Together, these complementary habitat characteristics may enhance biodiversity at the sandbar scale.

However, these spatial differences were strongly modulated by seasonal hydrological variation rather than remaining constant across seasons, suggesting that community structure within ICWBs is organized not by habitat type alone, but by the interaction between spatial context and temporal hydrological dynamics [28,55]. Rather than emphasizing any single habitat type or taxonomic group, these findings highlight the interaction between spatial context and temporal hydrological dynamics as a central organizing principle of ICWB communities. Disturbance-driven resetting of local assemblages, together with stochastic colonization and season-specific environmental filtering, may further contribute to these patterns [29,56].

From a broader river-ecosystem perspective, the present findings indicate that ICWBs on sandbars function as dynamic, context-dependent habitats whose ecological roles shift seasonally and spatially within the active channel. Rather than functioning as permanent or uniform refugia, their contribution appears to lie in maintaining habitat heterogeneity and temporal turnover within the river-channel habitat mosaic. This interpretation is consistent with riverine ecosystem synthesis frameworks emphasizing the importance of spatial–temporal habitat mosaics in maintaining biodiversity within river networks [3,11].

### Riverine ponds as dynamic nodes in a riverscape

RPs and other lentic or semi-lentic water bodies in riverine landscapes are often regarded as potential refugia for aquatic organisms during periods of high disturbance because of their reduced hydraulic stress and relative hydrological stability [e.g., 3, 6, 31]. However, our results did not support a consistent refugial function of RPs within ICWBs, despite this commonly proposed role [14].

The absence of taxa consistently associated with RPs across all seasons suggests that pond communities were repeatedly reorganized under changing environmental conditions rather than maintaining a stable core assemblage. Community composition shifted among seasons. Summer assemblages were characterized by opportunistic taxa such as Chironominae and multivoltine mayflies (e.g., *Cloeon* sp.) characterized by short life cycles and rapid responses to environmental fluctuation, whereas winter assemblages included overwintering and/or hyporheic taxa such as *Siphlonurus* spp. and Capniidae [32]. Other seasons included Coleoptera and Hemiptera together with hyporheic and lotic-associated taxa, reflecting seasonal changes in connectivity and environmental conditions [30,57].

These patterns suggest repeated community reassembly rather than directional succession toward a stable assemblage. Pond communities did not converge toward a single community state but instead shifted in response to changing environmental conditions and connectivity regimes over time. From a riverscape and metacommunity perspective, RPs on sandbars can therefore be viewed as temporally dynamic habitat nodes in which community composition is shaped by the interaction of seasonal connectivity, dispersal processes, and environmental filtering. These dynamics are consistent with conceptual frameworks emphasizing the role of patch turnover and spatial connectivity in structuring communities within dendritic river networks [31,58,59].

### Comparison between ICWBs and FWBs

Floodplain water bodies (FWBs) on high alluvial terraces are widely recognized as important aquatic–terrestrial ecotones maintained historically by recurrent flood pulses that support lateral connectivity and material exchange [1,3]. In regulated rivers, however, reduced flood frequency and magnitude often promote terrestrialization and riparian vegetation encroachment, accompanied by fine-sediment accumulation and habitat degradation as floodplain water bodies age [60,61]. Such changes can progressively reduce habitat suitability for aquatic organisms dependent on periodic connectivity with the main channel, including fish and bivalves [62,63].

Although FWBs were not investigated directly in the present study, comparison with the well-established conceptual framework for FWBs provides a broader context for interpreting the ecological roles of ICWBs in regulated rivers. In contrast, ICWBs on sandbars are renewed by more frequent but lower-intensity channel-scale disturbances, such that relatively small floods can restructure sandbar morphology and intermittently reset habitat conditions [21,24]. This disturbance regime promotes frequent habitat turnover while limiting long-term terrestrialization, thereby maintaining diverse in-channel habitat mosaics. Consistent with this interpretation, macroinvertebrate assemblages in ICWBs were clearly distinct from those in the main channel and contained many habitat-restricted taxa despite the transient nature of individual habitats.

Taken together, these contrasts suggest that ICWBs should not be viewed as substitutes for FWBs, but rather as complementary habitat components operating under different disturbance regimes and contributing to biodiversity in different ways. FWBs remain important for conserving floodplain biodiversity and maintaining lateral connectivity across river-floodplain interfaces, whereas ICWBs appear to contribute primarily through within-channel habitat heterogeneity and temporal turnover.

From a management perspective, this distinction is particularly important in regulated rivers, where large-scale floodplain reconnection is often socially or technically constrained [64,65]. Under such conditions, maintaining sediment mobility, sandbar dynamics, and channel features that promote ICWB formation may provide a feasible management pathway alongside floodplain restoration while supporting aquatic diversity within the active channel.

At the same time, this comparison remains conceptual and should be interpreted cautiously. Because the present study focused only on backwaters and RPs formed on a single sandbar within one regulated river reach, it was not designed to directly compare FWBs and ICWBs within the same system. Accordingly, our results support the hypothesis that ICWBs may complement the ecological functions of FWBs, but do not justify treating them as substitutes for floodplain restoration.

### Limitations

The present study was conducted at a single sandbar within one regulated river reach, and the spatial extent of inference is therefore limited. Accordingly, the observed patterns should be interpreted as site-specific evidence rather than as a general representation of all ICWBs in regulated rivers. Although the results provide insights into environmental gradients and macroinvertebrate community dynamics within ICWBs, caution is required when extrapolating these findings to river systems with different geomorphological settings, flow regimes, or management contexts. In addition, effect-size comparisons with the main channel were based on a single reference site and therefore represent relative contrasts to a consistent local reference rather than a full characterization of main-channel heterogeneity. Because spatial analyses were intended to describe directional gradients within a single sandbar, spatial autocorrelation among nearby sites was not explicitly modeled and may have influenced some observed spatial relationships.

In addition, the analyses were based on seasonal snapshots rather than continuous temporal trajectories, and thus short-term disturbance events and finer-scale temporal dynamics in hydrological connectivity and habitat turnover may not have been fully captured. Because habitat categories were defined at the time of each survey, transitions between backwaters and riverine ponds were incorporated only as seasonal states rather than continuously tracked habitat trajectories.

Finally, although the measured environmental variables explained a substantial portion of community variation, unmeasured factors such as substrate characteristics, organic matter accumulation, dissolved oxygen, shading, and stochastic colonization processes may also contribute to community assembly within ICWBs. These limitations do not alter the overall conclusion that ICWBs on sandbars function as spatially and temporally dynamic habitats, but they do indicate that the relative importance of specific drivers should be interpreted cautiously and evaluated further across a wider range of systems.

Because Seigyu structures of different installation ages co-occurred within the study sandbar, the present study was not designed to explicitly evaluate installation-age effects. Future studies explicitly comparing Seigyu structures of different installation ages may help clarify how channel-engineering history influences ICWB development and associated biodiversity patterns.

## Conclusion

The present study suggests that ICWBs function as highly dynamic habitats structured by the interaction between seasonal hydrological regimes and fine-scale spatial heterogeneity within sandbars. Macroinvertebrate communities differed among UBs, DBs, and RPs, reflecting a gradient from relatively stable to more disturbance-prone environments even within a single sandbar. Rather than serving as persistent refugia, these habitats appear to contribute to riverine biodiversity primarily through temporal turnover and the maintenance of heterogeneous habitat mosaics. Within this habitat mosaic, UBs, DBs, and RPs played distinct but seasonally variable ecological roles, with habitat function maintained through shifting community composition rather than stable occupation by fixed taxa. Consequently, in regulated rivers where lateral connectivity is constrained, maintaining sandbar dynamics and the processes generating diverse ICWBs may provide a complementary within-channel approach to supporting riverine biodiversity. Although this implication remains conceptual and system-specific, it highlights the potential value of conserving dynamic in-channel habitat mosaics alongside broader floodplain restoration.

## Supporting information

S1 Fig Longitudinal and lateral gradient of environmental variables within ICWBs on the sandbar. Results of generalized additive models against longitudinal distance from the upstream edge of the sandbar (a) and lateral distance from the water’s edge (b). EC, chl. *a* and FPOM were log_10_-transformed based on the results of Shapiro-Wilk normality tests. Green boxes indicate the variables showing significant relationships (P < 0.05). Colors indicate ICWB categories: upstream backwater (blue), riverine pond (red), and downstream backwater (green).
(TIFF)

S2 Fig Variation partitioning analysis of macroinvertebrate community composition within ICWBs. Variation partitioning was conducted using the varpart function in the vegan package to explore the relative contributions of environmental and spatial variables to community variation. Blue and pink areas represent fractions explained by environmental and spatial variables, respectively.
(TIFF)
